# Role of Bioactives in Neurodegenerative Diseases

**DOI:** 10.3390/ijms25094951

**Published:** 2024-05-01

**Authors:** Lefteris C. Zacharia

**Affiliations:** 1 Department of Health Sciences, School of Life and Health Sciences, University of Nicosia, 2417 Nicosia, Cyprus; zacharia.l@unic.ac.cy; 2 Bioactive Molecules Research Center, School of Life and Health Sciences, University of Nicosia, 2417 Nicosia, Cyprus

Neurodegenerative diseases (NDs) affect millions worldwide, with the two most prevalent being Alzheimer’s and Parkinson disease. The pathophysiology of these diseases is complex and is characterized by neuronal death ultimately affecting neural transmission and/or synapses, culminating in specific disease-related symptoms [1]. Besides their complex pathophysiology, the treatment of NDs is further complicated by the fact that drugs have to cross the blood–brain barrier, adding another level of complexity. While for Parkinson disease, a number of symptom-relieving drugs are available, for Alzheimer disease (AD), only a handful of symptomatic treatments are available with moderate efficacy, and these are effective only at certain stages of the disease. What’s more, there is an urgent need for effective disease-modifying drugs for NDs, as the number of affected individuals is projected to increase. The incidence of AD patients, for example, is increasing at an alarming rate and estimated to double every 20 years. According to the 2022 World Alzheimer report [2], in 2020, 55 million were affected, a number that is expected to increase to 78 million in 2030 and 139 in 2050. 

Despite the fact that β amyloid (Aβ) still remains an active hypothesis and target in AD, other emerging targets are gaining momentum, and many clinical trials are being planned. In 2023, there were 187 agents in clinical trials assessing 141 unique treatments. Across all phases, 79% were disease-modifying drugs, and the remaining 21% accounted for symptomatic drugs. Interestingly, 28% of these trials examine repurposed drugs. From the target perspective, 16% target Aβ, 9% target tau, 17% inflammation, 13% synaptic plasticity/neuroprotection, 7% metabolism and bioenergetics, 5% oxidative stress, and 3% proteinopathy [3,4].

Despite all these attempts, clinical trial failures are partly attributed to the timing of drug intervention, yet clinical trials remain the only way to test efficacy and safety of drugs for AD, as in vivo data do not usually predict efficacy for AD. Importantly, for complex neurodegenerative diseases, multi-target drug ligands or combination therapies are likely to be more effective with anti tau and anti Aβ combinations being currently investigated [5,6,7]. Finally, with all the novel therapies being investigated, along with the wealth of information gained so far from clinical trials, it is the hope that all these data will be integrated to develop a better understanding of how to tackle NDs.

The Special Issue in “Novel targets in neurodegenerative diseases” has hosted a number of research and review papers that have given new insights into the bioactives in neurodegenerative diseases, particularly Alzheimer’s and Parkinson disease. Most of the papers submitted deal with Alzheimer disease, which is consistent with the urgency for finding new drugs for this disease. The contributions referred to below add new insights to the NDs novel therapeutic approaches including natural compounds with cognitive enhancing effects.

## Contributions

In the contribution by De La Dorre et al, the in vivo efficacy of a high-affinity acyl-CoA:cholesterol acyltransferase 1 (ACAT1) inhibitor, termed F12511, delivered IV as a nanoparticle was tested. The results showed that a 2-week treatment of aging 3xTg AD mice ameliorated amyloidopathy, reduced both hyper- and non-phosphorylated tau, and reduced neuroinflammation. Importantly, F12511 has passed phase I trials as an antiatherosclerotic drug. Its efficacy in this in vivo study paves the way for further studies, and opens avenues for repurposing drugs for AD, consistent with the current trials of AD where repurposed drugs account for 28% of all the trials [3].

The study by Stańczykiewicz et al. tested ovocystatin, a cysteine protease inhibitor isolated from egg white, for its in vitro ability to inhibit Aβ aggregation. The results showed that ovocystatin has Aβ anti-aggregation activity and inhibits Aβ oligomer toxicity in neuronal PC12 cells. This contribution is in line with current therapies in AD as described by Cummings et al. [3], targeting Aβ, which account for 16% of the clinical trials. Despite the fact that, recently, studies have shifted their focus from Aβ, it is evident that many drugs are still directed against Aβ, thus valuing its involvement in AD.

Along the same lines of testing natural compounds but using in vivo evidence, Kochekova et al. investigated the effects of soybean lecithin and plasmalogens after being administered in the diet of Wistar rats for six weeks on short- and long-term memory, cognitive abilities, and grip strength. Lecithin enhanced memory and cognitive functions, while plasmalogens significantly improved appetite and increased grip strength. Lecithin significantly raised HDL levels while lowering LDL levels. The study’s findings imply that soy lecithin and plasmalogens may both be significant nutritional components for enhancing cognitive functions. 

Wen Meng et al. contribution dealt with another natural compound Paeoniflorin, a glycoside with antioxidant, anti-inflammatory and cognitive enhancing effects. In the study, its effect against memory loss and cognitive decline were tested in lipopolysaccharide (LPS)-induced mice model of neurobehavioral dysfunction. Treatment with paeoniflorin alleviated LPS-induced neurobehavioral dysfunction, as assessed by behavioral tests (T-maze, novel-object recognition, and Morris water maze tests). Furthermore, while LPS stimulated the amyloidogenic pathway-related proteins, paeoniflorin decreased this pathway at the protein level, suggesting that it may be useful in the prevention of neuroinflammation related to AD. 

Inyang et al. reviewed the effect of Capsaicin, a natural spicy-tasting compound found in chili peppers, with anti-inflammatory, antioxidant, and possible neuroprotective properties. Capsaicin intake has been associated with greater cognitive function in humans, and attenuating aberrant tau hyperphosphorylation in a rat model of AD. In this contribution, the evidence from rodent and/or cell culture studies were reviewed. Of the 11 selected studies, ten studies showed that capsaicin attenuated tau deposition, apoptosis, and synaptic dysfunction, but had conflicting effects on amyloid processing, and was not found to be an effective antioxidant. Eight studies where rodents were treated with capsaicin showed improved spatial and working memory, learning, and emotional behaviors. Overall, capsaicin showed promise in improving AD-associated cognitive, and behavioral changes in cellular and animal models, opening avenues for studying further its role in AD. 

The contribution by Kousparou et al. highlights the importance of polyunsaturated fatty acids in neurodegenerative diseases. The beneficial effects of eicosapentaenoic acid (EPA) and docosahexaenoic acid (DHA) polyunsaturated fatty acids (omega-3 PUFAs) have been reviewed in NDs as they play a role in suppression of inflammation, gene expression, and immune functionality, ultimately improving cognitive function. On the other hand, the role of omega-6 polyunsaturated fatty acids (linoleic acid-LA, gamma linolenic acid-GLA, and arachidonic acid-AA) on neuroprotection is controversial. The review provides an overview of the existing recent clinical studies with respect to the role of omega-3 and omega-6 PUFAs as therapeutic agents in NDs. 

Mlakic et al.’s study deals with novel compounds for the symptomatic treatment of AD. Novel compounds were synthesized and tested for their ability to inhibit cholinesterase, the target enzyme for the symptomatic treatment of AD. Specifically, a number of thienobenzo/naphtho-triazoles were synthesized, resulting in a large group of molecules with different functionalities in the structure, and the IC50 in butyrylcholinesterase and Acetylcholinesterase were evaluated, along with follow-up molecular modelling analysis and interpretation. The study brings new insight for the future design of thienobenzo/naphtho-triazole-based cholinesterase inhibitors as therapeutics for neurological disorders.

Nhieu et al. using a P19-motor neuron (MN) differentiation system identified a cellular retinoic acid binding protein 1 (CRABP1), termed C32, that lacks retinoic acid receptor (RAR) activity. CRABP1 has been identified as a new therapeutic target, especially for motor neuron degenerative diseases. C32 and C4 CRABP1 ligands were protective against excitotoxicity-triggered MN death. The study provides insights for the potential use of CRABP1-binding, at RA-like ligands in mitigating MN degenerative diseases. Importantly, these ligands are free of RAR activity that lead to toxic effects, paving the way for the study of new therapeutics for neurodegenerative diseases.

The contribution by Pacossi et al. dealt with PD and reviewed the genetic and epigenetic alterations in PD and the potential thereof as therapeutics especially when it comes to miRNAs, encapsulated in exosomes for the treatment of PD. Specifically, modified exosomes as delivery systems of bioactives including drugs or RNAs can be used to deliver them in the brain overcoming the blood–brain barrier, opening new avenues for PD therapeutics. 

Schneider Jay’s paper reviews the reasons behind failures of PD therapeutics as disease modifying agents, focusing on drugs with a single mechanism of action, and suggesting that an alternative successful strategy could be the use of multi-functional therapeutics that target multiple pathogenic mechanisms. Evidence is presented that the multi-functional glycosphingolipid GM1 ganglioside may be just such a therapeutic. This is in line with other evidence in the literature that suggests that for complex neurodegenerative diseases, multi-target drugs are more likely to have an effect [5,7].

Finally, Beliakov et al.’s contribution focuses on methods for neuroprotection as a means of increasing the efficacy of symptomatic pharmacotherapy aiming at restoring dopamine levels. In this regard, a relevant system such as dopaminergic cell line is essential for screening for neuroprotectors. To assess the mechanisms of neurodegeneration and neuroplasticity under the influence of toxins and antiparkinsonian drugs, including neuroprotectors, Beliakov et al. have developed Lund human mesencephalic (LUHMES) cell based 1-methyl-4-phenylpyridinium iodide (MPP+) PD model and proposed an original panel of markers for testing functional and structural cell disorders.

In conclusion, this Special Issue on the role of bioactives in NDs indicates that while new therapeutics are still being developed, natural compounds are also actively being investigated as a potential means of enhancing memory and alleviating disease-related symptoms. Some of the contributions complement the mainstream efforts in NDs and particularly ADs, while a number of contributions have emphasized the role of natural compounds in enhancing memory with the aim of being investigated further to complement the current knowledge, and as an alternative research direction. Ultimately, these could be combined with other emerging therapies for increasing symptom alleviation and/or disease progression.
